# Crosstalk between DnaA Protein, the Initiator of *Escherichia coli* Chromosomal Replication, and Acidic Phospholipids Present in Bacterial Membranes

**DOI:** 10.3390/ijms14048517

**Published:** 2013-04-17

**Authors:** Rahul Saxena, Nicholas Fingland, Digvijay Patil, Anjali K. Sharma, Elliott Crooke

**Affiliations:** 1 Department of Biochemistry and Molecular & Cellular Biology Georgetown University Medical Center, Washington, DC 20007, USA; E-Mails: dap89@georgetown.edu (D.P.); anjali0623@gmail.com (A.K.S.); crooke@georgetown.edu (E.C.); 2 Jet Propulsion Laboratory, California Institute of Technology, M/S: 183-426, 4800 Oak Grove Drive, Pasadena, CA 91109, USA; E-Mail: nick.k.fingland@jpl.nasa.gov; 3 Lombardi Comprehensive Cancer Center, Georgetown University Medical Center, Washington, DC 20007, USA

**Keywords:** acidic phospholipids, DnaA protein, chromosomal replication, *Escherichia coli*

## Abstract

Anionic (*i.e*., acidic) phospholipids such as phosphotidylglycerol (PG) and cardiolipin (CL), participate in several cellular functions. Here we review intriguing *in vitro* and *in vivo* evidence that suggest emergent roles for acidic phospholipids in regulating DnaA protein-mediated initiation of *Escherichia coli* chromosomal replication. *In vitro* acidic phospholipids in a fluid bilayer promote the conversion of inactive ADP-DnaA to replicatively proficient ATP-DnaA, yet both PG and CL also can inhibit the DNA-binding activity of DnaA protein. We discuss how cellular acidic phospholipids may positively and negatively influence the initiation activity of DnaA protein to help assure chromosomal replication occurs once, but only once, per cell-cycle. Fluorescence microscopy has revealed that PG and CL exist in domains located at the cell poles and mid-cell, and several studies link membrane curvature with sub-cellular localization of various integral and peripheral membrane proteins. *E. coli* DnaA itself is found at the cell membrane and forms helical structures along the longitudinal axis of the cell. We propose that there is cross-talk between acidic phospholipids in the bacterial membrane and DnaA protein as a means to help control the spatial and temporal regulation of chromosomal replication in bacteria.

## 1. Introduction

**Figure 1:**
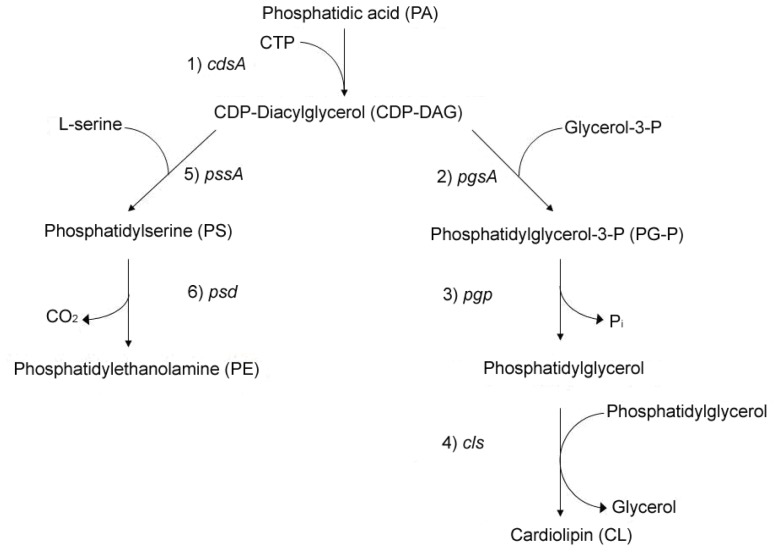
Biosynthesis of phospholipids in *Escherichia coli*. The synthesis of phospholipids is carried out in the steps as indicated and is catalyzed by the following enzymes that are encoded by the genes denoted at each step: (1) CDP-diacylglycerol synthase; (2) phosphatidylglycerolphosphate synthase; (3) phosphatidylglycerolphosphate phosphatase; (4) cardiolipin synthase; (5) phosphatidylserine synthase; (6) phosphatidylserine decarboxylase.

*Escherichia coli* inner membrane contains a mixture of phospholipids with a composition of approximately 70% phosphatidylethanolamine (PE), 25% phosphatidylglycerol (PG) and ~5% cardiolipin (CL), with a small remaining fraction of metabolic intermediates [1]. The precursor of these phospholipid species is CDP-diacylglycerol (CDP-DAG). CDP-DAG can then be shunted through two pathways to make either zwitterionic PE or the acidic PG and CL (Figure 1). In one pathway, addition of serine to CDP-diacylglycerol via phosphatidylserine synthase (PssA) results in phosphatidylserine (PS), which subsequently get decarboxylated by phosphatidylserine decarboxylase to form PE [2–5]. Alternatively, transfer of glycerol-3-phosphate onto CDP-diacylglycerol, predominantely by phosphatidylglycerophosphate synthase (PgsA), followed by a subsequent dephosphorylation by phosphatidylglycerophosphate phosphatase (Pgp), leads to the synthesis of PG [3,5]. Two molecules of PG condense to form CL through the action of cardiolipin synthase (ClsA) [6–8].

Anionic phospholipids are ubiquitous in nature. For example, PG and CL are associated with the photosystem II complexes of higher plants [9–11]. CL has been shown to be present in bacterial membrane [12–14], mitochondrial inner membrane [14–17], and the hydrogenosome membrane of anaerobic protist and fungi [18]. Besides, serving as a component of membrane bilayer, acidic phospholipids appear to regulate several critical cellular functions via protein-lipid interactions governed by various mechanisms, such as ion-mediated salt bridges [19] and electrostatic interaction [20–23]. These functions include acidic phospholipid induced (particularly CL) apoptosis in mitochondria [24–26], oxidative phosphorylation [27,28], and regulation of respiratory complexes in bacteria [29,30] and yeast [31]. The interaction of CL with Lon protease, which is involved in degrading misfolded proteins [32,33] influences the action of Lon by inhibiting its proteolytic and ATPase activities [34].

In prokaryotes, the role of acidic phospholipids also appears to be linked to chromosomal and cell division-related events [35] including the initiation of chromosomal DNA replication [36–39]. *In vivo* evidence links proper cellular levels of PG and CL with continued cell growth [40,41] and normal chromosomal replication [36–39], in that reduced levels of acidic phospholipids, arising from repressed expression of *pgsA*, result in arrested-growth and inhibited chromosomal replication in otherwise wild-type *E. coli*.

Growth of *clsA* mutants is affected to a lesser extent than that for *pgsA* mutants [40–42]. This in part may be due to the cells possessing redundant pathways for CL synthesis [7,43]. *Bacillus subtilis* mutants lacking the *clsA* gene still possess CL domains that appear after sporulation is initiated [44]. *E. coli* defective for ClsA activity also appear to maintain residual levels of CL, with PssA implicated in its formation possibly by donating a phosphatidyl group to glycerol [7]. Moreover, other genes homologous to *cls* have been identified in *E. coli*. One, named as *clsB*[45], also known as *f413* or *ybhO*[43,45] encodes for a protein with the characteristic feature of having HKD motifs found in the phospholipase D (PLD) protein superfamily, which also includes cardiolipin synthase [45]. Biochemical characterization of the protein translated from *E. coli clsB* reveals that although kinetically less active, the protein can catalyze the formation of CL from PG. Thus, the possibility cannot be excluded that the *clsB* gene product can generate enough CL to support essential CL-dependent functions in *cls*A null cells. Moreover, a recent study has identified a third *cls* homologue, termed *clsC*, which also contains HKD motifs [43]. Unlike the *clsA* gene product, the protein encoded by *clsC* uses PE as the phosphatidyl donor to PG for the formation of CL, and does so in a manner dependent on coexpression of the *ymdB* gene that precedes *clsC* in an operon. A triple *cls*ABC mutant has been shown to lack any detectable level of CL and has reduced viability when in the stationary phase [43].

A large body of *in vitro* and *in vivo* data indicates that the action of DnaA protein as the initiator of chromosomal replication is modulated by PG and CL residing in the fluid bilayer of the bacterial inner membrane [46–48]. Acidic phospholipids have the ability to promote the exchange of the tightly bound allosteric effectors ADP and ATP (see sections 3), and studies have shown that acidic phospholipids can inhibit the formation of the replicatively active nucleoprotein complex at the origin of chromosomal replication (*oriC*) in *E. coli*[49,50] (see section 7). The molar ratios of membrane phospholipids appears to change as cells pass from exponential growth into stationary phase [51,52] and recent work shows that depletion of cellular acidic phospholipids leads to under initiation of replication from *oriC* during the cell-cycle [39].

Acidic phospholipids, particularly CL, are present in the form of lipid domains that can be visualized using the CL-specific fluorescent dye *10-N-nonyl acridine orange* (*NAO*) [12,15]. These domains are localized at negatively-curved regions of bacterial cell membranes [53]. The role of acidic phospholipids in directing membrane curvature has been the focus of studies, as it has their role in the localization of various proteins, such as the cell division protein MinD [53–55], the osmosensory transporter, ProP [56–58] and the SecYEG protein complex [59,60]. With studies showing DnaA localized at the plasma membrane [61,62], the spatial arrangement of DnaA with respect to acidic phospholipid domains will be an interesting aspect to examine.

Based on acidic phospholipids affecting the nucleotide-bound state of DnaA, the ability of DnaA to productively bind to *oriC*, and the localization of DnaA at the cell membrane, we propose that there is cross-talk between the *E. coli* chromosomal initiator protein, DnaA, and acidic phospholipids present in the bacterial membrane. A review of supporting literature is presented below.

## 2. Linkage between Bacterial Growth and Membrane Acidic Phospholipids

Intriguing observations suggest that the total cellular anionic lipid content present in the membrane influences bacterial growth [40,41]. Growth of the bacterial cells harboring a sole chromosomally-encoded copy of the *pgsA* gene under an inducible promoter can be regulated by the absence or presence of the inducer in the medium [41]. When grown in the absence of the inducer, the cells continue to grow for several generations until the levels of PG and CL decrease to threshold amounts, at which point the cells undergo a growth-arrest. The arrested cells remain viable, and if *pgsA* expression is again induced, the cells resume growth shortly afterwards [41].

Interestingly, the growth-arrest of acidic phospholipid-depleted cells can be bypassed. One such mechanism is when bacterial cells can grow in the presence of otherwise insufficient levels of cellular acidic phospholipids because they possess mutations in *rnhA*[36], which encodes for RNaseH that degrades RNA within RNA-DNA hybrids [63,64]. In contrast to normal DnaA protein-dependent replication initiation from *oriC*, these cells, in a RecA-dependent manner, use persistent RNA-DNA hybrids formed in absence of RNaseH to serve as sites for the initiation of DNA synthesis, a process termed constitutive stable DNA replication (cSDR) [63,64].

In *E. coli*, the growth-arrested phenotype of a *pgsA* deletion also can be reversed if the cells lack *lpp*, the gene encoding Lpp (murein lipoprotein), a major outer membrane lipoprotein [65]. Biosynthetic maturation and translocation of Lpp from the inner to outer bacterial membrane involves the transfer of the diacylglyceryl moiety from PG to cysteine-21 of prolipoprotein, producing the diacylglyceryl modified intermediate, DGPLP [66,67]. Blocking the diacylation of prolipoprotein by either lack of phosphatidylglycerol due to repressed *pgsA*, or by introducing a cysteine-21 to glycine point mutation results in accumulation of unmodified, immature protein product (UPLP) in the inner cell membrane, coincident with reduced viability [66,68]. The defect in bacterial cell growth has been attributed to an anomalous covalent linkage between accumulated UPLP and peptidoglycan at the cell membrane [68]. However, another hypothesis yet to be addressed is whether accumulation of UPLP may also adversely affect *oriC*-dependent DNA initiation.

**Figure 2:**
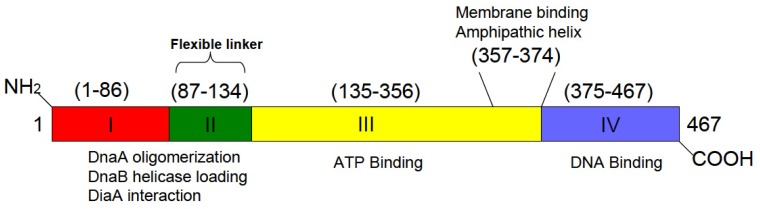
Schematic representation of DnaA protein domains. DnaA protein has four distinct functional domains. Domain I, comprising amino acid residues 1–86, and flexible linker region domain II (87–134) are involved in protein-protein interaction. Domain III (135–356) contains conserved features of the AAA + protein superfamily and is involved in ATP binding. The *C*-terminus of domain III features an amphipathic helix (357–374), which is responsible for DnaA binding to acidic membranes. Domain IV (375–467) is essential for DNA binding and nucleoprotein complex formation.

DnaA protein initiates chromosomal replication at *oriC* once per cell cycle. A third mechanism to suppress the growth arrest of acidic phospholipid-deficient cells, besides harboring the *rnhA* genetic background that allows cSDR to occur, or cells having a *lpp* null mutation, is over-expression of DnaA protein possessing certain deletion and point mutations in its membrane-binding or DNA-binding domains (Figure 2) [37]. One well characterized mutant form of DnaA is DnaA(L366K), which can restore growth to acidic phospholipid-depleted cells [37,39].

Biochemically, DnaA(L366K) is similar to wild-type in several properties, including nucleotide binding and hydrolysis (see sections 2 and 3). Yet, DnaA(L366K) can initiate replication only when a limited amount of wild-type DnaA is present [69]. In agreement, nucleoprotein complexes (see section 2) containing only DnaA(L366K) protein were found inefficient at unwinding DNA duplex at *oriC*, and thus are unable to independently support DNA synthesis [70]. However, mixed oligomers containing DnaA(L366K) along with wild-type DnaA form productive nucleoprotein complexes [70]. The *N*-terminal domain of DnaA protein is responsible for oligomerization of DnaA-DnaA protomers (Figure 2) [71–73]. Therefore, mutations present either in the membrane binding domain or *C*-terminus DNA binding domain of DnaA protein might not affect formation of mixed, but functional heteroligomers between wild-type and the mutant forms of DnaA.

Limited structural data on DnaA protein in different bacteria provide a major challenge to understanding what conformational changes in DnaA(L366K) or mutant forms of DnaA with certain small, internal deletions in the *C*-terminal region allow over-expression of the mutant proteins, but not the wild type protein, to bypass the arrested-growth phenotype. However, findings from a study that used an ATP fluorescent analog, 2′-(or-3′)-*O*-(*N*-methylanthraniloyl) adenosine 5′ tri-phosphate (MANT-ATP), suggest that DnaA(L366K) might require a lower concentration of acidic phospholipids to induce the exchange of ADP to ATP bound to DnaA protein [74]. This study postulates that the low levels of PG and CL arising from repressed *pgsA* expression may be sufficient for promoting ADP-ATP exchange on DnaA(L366K), but not wild-type DnaA.

## 3. Importance of the Nucleotide State of DnaA Protein in Determining the Functional Status of Nucleoprotein Complex Generated at *oriC*

For several decades DnaA has been known to be an essential protein involved in chromosomal replication [75–77]. Formation of a productive nucleoprotein complex of DnaA protein bound to *oriC* causes DNA conformational changes that trigger melting of nearby duplex DNA [77–79]. This is followed by DnaA-mediated recruitment of DnaB helicase to sites of the future replication forks at the melted double-stranded DNA, and ultimately the assembly of replisomes that will carry out bi-directional chromosomal replication [80–82].

**Figure 3:**
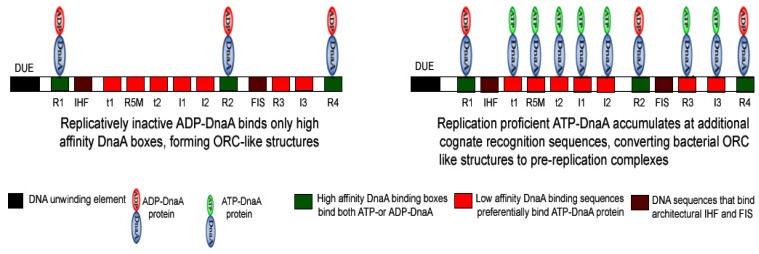
Schematic representation of *oriC: Escherchia coli oriC* (approximately 250 base pairs) contains cognate recognition sequences for DnaA protein. Based on their affinity for ADP-DnaA and ATP-DnaA these DNA elements are categorized as high affinity (R1, R2 and R4) and low affinity (R5M, I1, I2 I3, I4, tau 1 and tau 2 sites) DnaA binding sites. Binding of ADP-DnaA and ATP-DnaA to high affinity DnaA binding elements form an ORC. At the onset of chromosomal replication, the ORC is converted to a pre-RC by oligomerization of additional DnaA protein molecules to occupy low affinity DnaA binding sites.

In binding to the approximately 250 base pair region of *oriC*, several molecules of DnaA protein interact with multiple asymmetric DnaA-binding sequences, termed as R boxes [76,83], I sites [84], and τ sites [85]. DnaA tightly associates with the adenine nucleotides ATP and ADP (*K*_D_ of 0.03 and 0.2 μM, respectively) [77]. However, whether ATP or ADP is tightly bound to DnaA protein, determines DnaA protein’s preferential binding to specific *oriC* elements. The binding of ADP-DnaA or ATP-DnaA to R1, R2, and R4 boxes constitutes an origin recognition complex (ORC) (Figure 3) [70,86,87], which persists throughout most of the cell-cycle [88]. In contrast, low affinity I sites (I1, I2, I3 and I4) [70,84] and τ sites (τ1 and τ2) [85] show preferential binding by only ATP-DnaA to generate a pre-replication complex (pre-RC) (Figure 3) [70,86,87]. The engagement of low affinity sites I2 and I3 with ATP-DnaA is required for the progression from an ORC to a pre-RC and DNA strand opening (Figure 3). Recently, it has been shown that at the time of initiation, DnaA protein extends the assembly from the high affinity to low affinity DnaA binding sites [87].

## 4. Acidic Phospholipids Promote Conversion of Replicatively Feeble ADP-DnaA to the Replicatively Active ATP-Form

**Figure 4:**
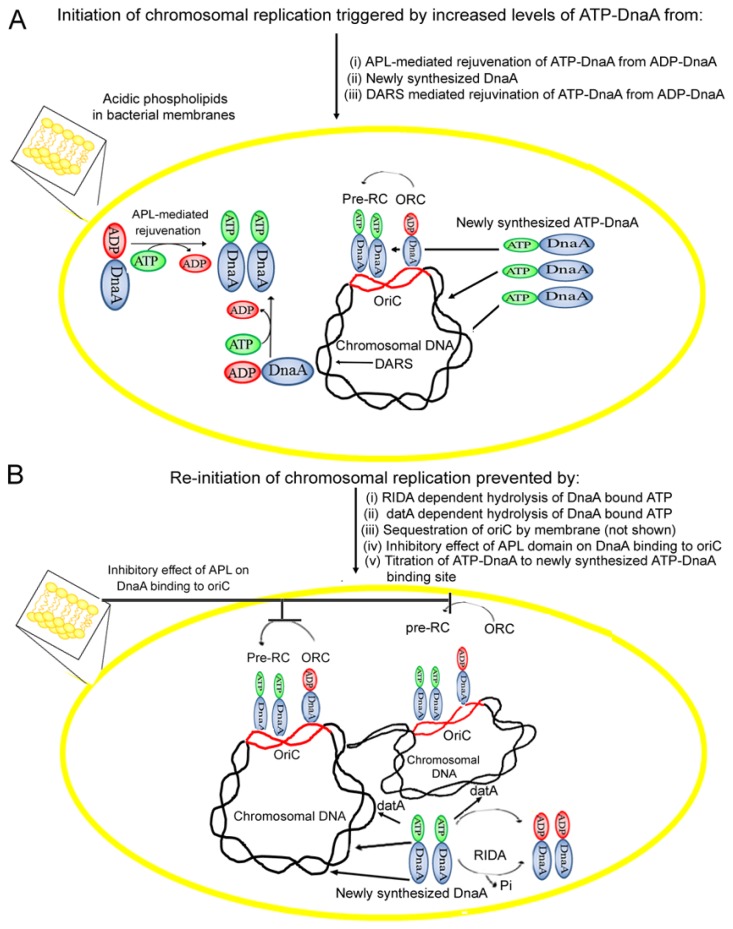
The cross talk between acidic phospholipids (APL) and DnaA. (**A**) Prior to initiation, inactive ADP-DnaA occupies high-affinity sites on *oriC* to form an ORC. As active ATP-DnaA concentration increases through acidic phospholipid stimulated DnaA exchange of ADP-ATP, DARS, and synthesis of new DnaA protein, low affinity DnaA binding sites in *oriC* are filled, and chromosomal replication is initiated; (**B**) After initiation, a combination of mechanisms to prevent re-initiation (i) RIDA (ii) sequestration of DnaA (iii) inhibition of DnaA binding to *oriC* by acidic phospholipid domains (iv) tritration of ATP-DnaA by other DnaA binding loci present at chromosome, such as *datA*, ensure initiation only occurs once per cell-cycle. Conversion of ADP-DnaA to ATP-DnaA may also occur through interaction with DARS.

One mechanism in *E. coli* to ensure that initiation occurs only once per cell-cycle is known as Regulatory Inactivation of DnaA (RIDA), which promotes the hydrolysis of DnaA-bound ATP, and thus the conversion of replicatively active ATP-DnaA to inactive ADP-DnaA, a process that involves Hda protein (homologous to DnaA) [89,90]. The hydrolysis of replicatively active ATP-DnaA to inactive ADP-DnaA is stimulated by the interaction of ATP-DnaA with ADP-Hda protein via inter-protein domain interactions [91,92]. It has been shown earlier that the cellular levels of ATP-DnaA are tightly controlled by Hda activity, as cells lacking *hda* gene, result in over-initiation of chromosomal replication [93,94]. Besides RIDA, hydrolysis of ATP-DnaA has also been attributed to another chromosomal locus, *datA*, previously described as reservoir for DnaA protein molecules, to prevent untimely initiation in a manner dependent on nucleoid-associated integration host factor (IHF) [95,96]. The resulting drop in the cellular concentration of ATP-DnaA, coupled with the synthesis of new DnaA-binding sites as genome duplication proceeds, lowers the initiation potential of DnaA protein below a needed threshold, and thereby preventing the re-initiation of replication during the same cell-cycle (Figure 4).

For the next round of chromosomal replication to occur in daughter cells, the initiation potential of DnaA must again rise above a certain threshold. Increased ATP-DnaA can occur through regeneration of ATP-DnaA from inactive ADP-DnaA in combination with *de novo* DnaA synthesis [97] (Figure 4). The *in vitro* exchange of bound ADP for ATP on purified DnaA is slow relative to the time of the bacteria cell cycle, with only half of purified ADP-DnaA converted to ATP-DnaA after 40 min, even in the presence of excess ATP [77]. However, there are mechanisms capable of accelerating the rejuvenation of ADP-DnaA to ATP-DnaA (Figure 4).

*In vitro* incubation of *oriC*-bound ADP-DnaA and excess ATP in the presence of acidic phospholipids, such as PG or CL, results in rapid release of bound ADP and exchange for ATP [46–48]. CL is approximately 10-times more potent than other anionic lipids in promoting release of DnaA-bound nucleotide. In contrast, zwitterionic phospholipids, such as phosphatidylcholine (PC) and PE, fail to stimulate the release of DnaA-bound nucleotides [46,48,69]. Thus, acidic phospholipids have been proposed to catalyze the rejuvenation of ADP-DnaA to ATP-DnaA *in vitro*[46–48].

Membrane-catalyzed nucleotide dissociation from DnaA protein is regulated by the DnaA-to-phospholipid ratio present on the membrane. In fact, two different functional states of DnaA protein exists at high and low membrane occupancy, which influences the release of nucleotide from protein [98]. Using the fluorescent ATP derivative MANT-ATP, it has been shown that crowding of DnaA protein on membrane could be induced by changes in temperature or the presence of Ficoll as a crowding agent [98].

The role of specific intergenic sequences, known as DnaA reactivating sequences, or DARS, has also been shown to promote rejuvenation of ADP-DnaA to ATP-DnaA, independent of CL [99,100]. However, cells deficient in acidic phospholipids, but possessing DARS are not able to grow [39–41]. Thus, DARS may not be the predominant or sole mechanism to carry out DnaA rejuvenation in the bacterial cells.

## 5. Membrane Fluidity Determines the Rate of ADP to ATP-DnaA Exchange

In addition to membrane lipids needing an acidic head group to promote the rejuvenation of DnaA, the bilayer must also be in the fluid phase. The fatty acid components of PG-containing small unilamellar vesicles has a strong impact on whether the vesicles are effective at releasing ADP from DnaA, with dipalmitoyl-PG approximately 10-fold less active than dioleoyl-PG [47,48]. Furthermore, phospholipids isolated from the bacteria lacking unsaturated fatty acids are feeble at promoting the exchange of DnaA-bound nucleotide [47,48]. This was seen by extracting lipids from *E. coli* treated with 3-decynoyl-*N*-acetylcysteamine (DNAC), an analog that interrupts the synthesis of unsaturated fatty acids and prevents the initiation of replication *in vivo*[101]. However, addition of oleic acid to the growth medium relieves the adverse effect of DNAC on growth, and phospholipids extracted from oleic acid-rescued DNAC-treated cells are active at promoting the rejuvenation of ADP-DnaA protein [47,48]. Moreover, phospholipids extracted from *fabA* mutant cells, which are defective in the synthesis of unsaturated fatty acids [101], grown in absence or presence of oleic acid vary significantly with respect to their fluidity and ability to dissociate DnaA-bound ADP [47,48]. Indeed, a tight correlation was observed between the degree of membrane fluidity, as measured by fluorescence anisotropy, and the extent that the membranes can stimulate nucleotide release from DnaA [47,48].

*E. coli* vary the fatty acid composition of membrane phospholipids with changes in temperature, thereby allowing the bacteria to modulate membrane fluidity in order to optimize cellular functions at different temperatures [102,103]. Suggestive that DnaA-mediated initiation of chromosomal replication is a function affected by membrane fluidity are observations that levels of unsaturated fatty acids are lower in cells harboring a *dnaA* temperature-sensitive allele than in wild-type cells at elevated temperatures [104], and conversely, the levels of unsaturated fatty acids are less in cells with a cold-sensitive *dnaA* allele that causes hyperinitiation than in wild-type cells at lower temperatures [104,105]. These changes in fatty acid composition have been proposed help stimulate the feeble initiation activity of the temperature-sensitive DnaA protein at higher temperatures and restrain the hyperinitiation activity of the cold-sensitive DnaA protein at lower temperatures, and that the changes in fatty acid composition occur through DnaA transcriptionally regulating the expression of proteins involved in fatty acid metabolism [104].

## 6. A Discrete Region of DnaA Associates with Fluid Bilayers Possessing Acidic Phospholipids

Immunofluorescence microscopy and immunogold labeling of cryothin sections with affinity purified anti-DnaA protein revealed that the majority of DnaA in a cell is localized at the plasma membrane, with approximately a 35-fold higher density in close proximity to the cell membrane than in the cytosol [61].

Two independent studies indicate that DnaA has a specific region that is responsible for its interaction with acidic membranes. In the first study, limited proteolytic digestion of DnaA with chymotrypsin and trypsin generated fragments of 35 kDa (residues D118–F458 of DnaA) and 29 kDa (residues S115–K372 of DnaA), respectively. Both fragments retained high-affinity for adenine nucleotides, yet only the larger chymotryptic fragment released bound nucleotide in response to treatment with acidic phospholipids in a fluid bilayer. Moreover, if DnaA was first allowed to associate with acidic membranes before treatment with trypsin, cleavage at K372 to generate the 29 kDa fragment was prevented, and instead a 30 kDa fragment (S115–K381) was obtained. The 30 kDa fragment, like the 35 kDa chymotryptic fragment and full-length DnaA, released its bound nucleotide when incubated with acidic phospholipids at a temperature that bestowed fluidity to the membrane bilayer. Thus, it is likely that the portion of DnaA near lysine 372 directly associates with acidic phospholipid bilayers [106].

Independently, crosslinking studies that utilized the photoactivable phospholipid analog 1-*O*-hexadecanoyl-2-*O*-[9-[2-[^125^I]iodo-4-(trifluoromethyl-3*H*-diazirin-3-yl)benzyl]oxy]carbonyl] nonanoyl]-*sn*-glycero-3-phosphocholine as a probe in acidic and neutral phospholipid bilayers provides additional evidence of a direct interaction between DnaA and acidic phospholipids. The study revealed that DnaA at the site of a putative amphipathic helix (amino acids 354–372) inserts into the hydrophobic interior of lipid bilayers only when the bilayer is enriched in acidic phospholipids and has the same degree of fluidity that promotes nucleotide exchange [107].

## 7. DnaA, Acidic Phospholipids, and Electrostatic Interactions

The requirement for the fluid bilayer to also have acidic headgroups appears to be due to the acidic head groups’ participation in the electrostatic recruitment of DnaA, a basic protein, to the lipid bilayer [20]. Such a mechanism is in agreement with the observation that even though *E. coli* lacks phosphatidylinositol (PI) or sphingolipids, negatively charged PI and ganglioside GM_1_ (monosialotetrahexosylganglioside) are equal to PG in their capacity to stimulate the release of DnaA-bound adenine nucleotide [46,106]. Notably, PG, PI and ganglioside GM_1_ have structurally distinct polar head groups. Furthermore, in contrast to ganglioside GM_1_, asialo-GM_1_ is feeble at promoting nucleotide exchange [106]. Perhaps not surprising, CL with its more anionic nature is significantly more potent in reactivating DnaA when compared to other acidic glycerophospholipids [46,106]. Phosphatadic acid (PA), another anionic phospholipid can also stimulate the exchange ADP to ATP over DnaA protein [48,106]. Interestingly, cells with little PG and CL have significantly higher levels of PA [65]. Together, these observations suggest that fluid bilayer’s enriched with acidic head groups is more important than any specific head group structure in promoting membrane-mediated ADP-ATP exchange on DnaA.

Supporting the importance of electrostatic forces for DnaA-membrane association, stable DnaA-lipid bilayer interaction is sensitive to ionic strength, as assessed by isopycnic centrifugation and intrinsic tryptophan fluorescence measurements [20]. Site-directed mutation analysis of DnaA structure-function revealed that basic residues Arg-360, Arg-364 and Lys-372 are indispensable for CL-mediated release of DnaA-bound nucleotide [108,109]. Of note, the region of DnaA (residues Asp-357–Val-374) containing these key residues is the same as that containing the proposed amphipathic helix involved in membrane binding of DnaA protein [106,107]. This prediction of an amphipathic helix in *E. coli* DnaA is supported by the solved crystal structure for a truncated form of *Aquifex aeolicus* DnaA [110]. Indeed, sequence comparisons show that these amino acids are well conserved among different bacterial species [110].

Examinations of additional point mutations revealed that the amino acids Arg-328, Arg-334 and Arg-342 present in another potential amphipathic helix (Lysine-327–Ile-345) also are important for DnaA-CL association [111]. Of these, Arg-328 and Lys-372 seems to be the most critical since CL interactions with these basic amino acid residues may change the confirmation of the ATP binding pocket, which further stimulates the release of ADP from the protein [112,113].

## 8. Acidic Phospholipids Inhibit DnaA Binding to *E. coli oriC* DNA

In addition to promoting exchange of DnaA-bound nucleotide, CL has been proposed to also inhibit DnaA binding to *oriC* (Figure 4). Filter retention assays demonstrated that nucleoprotein complexes of DnaA-*oriC* DNA remain intact when treated with PG or CL and ATP [49]. However, when nucleotide-bound or nucleotide-free DnaA is first treated with anionic phospholipids, the DnaA no longer is able to bind *oriC* DNA [49]. Thus, reactivation of ADP-DnaA to ATP-DnaA only occurs when DnaA, *oriC*, ATP, and anionic phospholipids in a fluid bilayer act in concert.

Interestingly, the degree that different phospholipids inhibit DnaA binding to *oriC* follows in a similar order to that observed for nucleotide exchange. CL was found to be the most potent in inhibiting DnaA-*oriC* interaction, whereas PG showed a 10-fold less inhibitory effect on the assembly of DnaA at *oriC*. Treating DnaA with neutral lipids, such as PC and PE, had little consequences for DnaA binding to *oriC*[50]. As with nucleotide exchange, the physical state of the bilayer influences the capacity of acidic phospholipids to inhibit DnaA-*oriC* interaction. Vesicles composed of di-linoleoyl PG inhibit the formation of DnaA-*oriC* nucleoprotein complexes more effectively than PG-liposomes with stearic acid acyl components [50].

Recently we observed that nucleotide-free DnaA protein exposed to liposomes of dilinoleoyl PG, CL or a mixture of phospholipids extracted from exponentially growing *E. coli* cultures abolishes DnaA binding to both high and low affinity DnaA sequences within *oriC*. On the other hand, nucleoprotein complexes formed in the presence of ATP or ADP remains unaffected by subsequent addition of purified acidic phospholipids as well as total lipids extracted from *E. coli* (unpublished result). These observations reflect the possibility that the ordered assembly of DnaA protein at specific recognition sequences might depend on whether DnaA is first exposed to cognate nucleic acid binding sites or to acidic phospholipids. Notably, the negatively charged polar head groups of acidic phospholipids and the negatively charged phosphodiester backbone of DNA may interact in a similar manner with basic proteins (Figure 4) [114,115], such as to preclude DnaA from binding to *oriC* when bound to the acidic phospholipids. If such negative control regulates DnaA binding to *oriC* to prevent re-initiation (Figure 4), it is unknown how this negative effect on DnaA activity is relieved or bypassed to allow normal initiations at the proper time in the cell-cycle. Additionally, the possibility cannot be excluded that cellular levels of acidic phospholipids might affect the binding of DnaA protein to cognate sequences other than *oriC*, such as, DARS and *datA*, due to similar nature DNA-binding interactions.

Studies have shown that total lipids isolated from exponentially growing *Staphylococcus aureus* are active in promoting the release of bound nucleotide from *S. aureus* DnaA protein, whereas lipid isolated from cells in stationary phase were inactive [116]. Of interest, earlier studies found that cellular lipid composition varies with the growth phase of *E. coli*, with a significant increase in CL levels as cells enter into stationary phase [51,52], perhaps in contrast to what one would expect for those lipids being active at promoting nucleotide release. Conversely, an increase of CL in stationary phase could be commensurate with the observed function of CL in inhibiting DnaA binding to *oriC.* Moreover, changes in lipid composition as cells move from one growth phase to another may differ between bacterial species.

## 9. Cardiolipin Helps Sub-Cellular Localization of Certain Bacterial Proteins

Acidic phospholipids, rather being homogenously distributed over the surface of bacterial cell membrane, exist as discrete domains at the poles and in the septal region of the cytoplasmic membrane of bacteria such as *E. coli*[12] and *B. subtilis*[44]. These domains-like structures can be visualized in living cells using the CL-specific fluorescent dye, *NAO*[12,15,44]. Unpublished observations suggest that the number and the location of CL-enriched domains in *E. coli* change as cells progress through the cell cycle.

*E. coli* having mutations in cytoplasmic division proteins form miniature cells (or mini cells) that lack DNA as a result of cell division occurring near the cell pole [117,118]. Examination of the membrane from minicells in *E. coli*[118] and forespore membranes in *B. subtilis*[44] produced during sporulation, showed enhanced levels of CL, which form domain-like structures.

The presence of certain lipids at regions of membrane curvature serve to target protein-lipid complexes to cell poles [53,56,58,119]. The cell division protein MinD that acts to inhibit septum Z-ring formation [120] preferentially binds to CL and localizes to the negatively curved regions of *E. coli* membranes [53]. CL is also shown to promote the polar location of other proteins besides MinD, such as *E. coli* osmosensory transporter ProP [56–58], and mechanosensitive channel protein MscS [58] and DivIVA in *B. subtilis*[121].

Whereas some protein are found concentrated at poles, there are proteins that form helices beneath the cell membrane, extending from pole to pole along the cell’s longitudinal axis, such as the cytoskeleton protein MreB and MinCDE [122,123]. Although *E. coli* DnaA, visualized by confocal microscopy of an internally-tagged GFP-DnaA fusion, also forms helical structures along the longitudinal cell axis, these helical structures exist distinct from and independent of MreB filaments [62]. Other studies using a chromosomally-encoded DnaA-EYFP protein did not detect a helical structure for DnaA [124], but this tagged protein was proposed [124] to be more active in initiation than the internally-tagged GFP-DnaA fusion, suggesting differences between the two proteins. Moreover, problems with photobleaching prevented visualization of the DnaA-EYFP protein by confocal microscopy [124]. A plasmid-born mcherry-tagged DnaA protein did not reveal helical structures, but that is not surprising given the high level of overexpression of mcherry-DnaA [125]. Interestingly, the overall global helical pattern formed by internally-tagged GFP-DnaA protein remains unaltered in the bacteria containing significantly reduced levels of acidic phospholipids (unpublished results), suggesting that localization of DnaA protein is not influenced by CL domains within the cell. Mechanisms that serve to regulate the spatial distribution of DnaA remain unknown.

## 10. Concluding Remarks

There is a wealth of data suggesting that the ability of DnaA to normally initiate *E. coli* chromosomal replication at *oriC* is influenced by acidic phospholipids present in the cell membrane. Depletion of the acidic phospholipids via repressed expression of *pgsA* results in under initiation of replication and arrested cell growth. The growth arrest phenotype can be relieved by abnormal initiations events at loci other than *oriC* or by expression of DnaA protein harboring a point mutation in its membrane-binding, both of which suggest a close link between cellular membrane composition and essential DnaA-mediated initiations at *oriC*. Of interest, preventing the accumulation at inner membrane of an intermediate of the murein lipoprotein (Lpp) synthesis pathway also can relieve the growth-arrest phenotype of acidic phospholipid-deficient cells, raising the question of whether the accumulated intermediate of Lpp synthesis adversely affects DnaA’s action at *oriC*.

Acidic phospholipids are concentrated in domain-like structures within the bacterial cell membrane, which change in a cell-cycle dependent manner. The acidic phospholipids may help determine the sub-cellular localization of proteins involved in cell division. It is worth asking might these anionic phospholipid domains also help dictate functional subcellular localization of DnaA protein bound to *oriC*, and are changes that occur to these domains during the cell-cycle somehow linked to the onset of replication of chromosomal DNA?

Acidic phospholipids PG and CL may participate in multiple critical cellular processes related to chromosomal replication. These include: (i) rejuvenation of ADP-DnaA to ATP-DnaA to support rounds of replication in subsequent cell-cycles; (ii) inhibition of DnaA binding to *oriC* to help set the precise timing of when DNA synthesis occurs; and (iii) possibly helping define the subcellular localization of chromosomal replication components. As such, we hypothesize crosstalk between DnaA protein and acidic phospholipids.

## Abbreviations

PG: Phosphatidylglycerol
CL: cardiolipin
*E. coli*: *Escherichia coli*
PE: phosphatidylethanolamine
CDP-DAG: cytosine diphosphate diacylglycerol
PssA: phosphatidylserine synthase
PS: phosphatidylserine
PgsA: phosphatidylglycerophosphate synthase
Pgp: phosphatidylglycerophosphate phosphatase
ClsA: cardiolipin synthase
*B. Subtilis*: *Bacillus subtilis*
PLD: phospholipase D
*oriC*: origin of chromosomal replication
*NAO*: 10-*N*-nonyl acridine orange
cSDR: constitutively stable DNA replication
MANT-ATP: 2′-(or-3′)-*O*-(*N*-methylanthraniloyl) adenosine 5′ tri-phosphate
ORC: origin recognition complex
pre-RC: pre-replication complex
RIDA: regulatory inactivation of DnaA
PC: phosphatidylcholine
DARS: DnaA reactivating sequences
DNAC: 3-decynoyl-*N*-acetylcysteamine
PI: phosphatidylinositol
GM_1_: monosialotetrahexosylganglioside
PA: Phosphatadic acid
*S. aureus.*: *Staphylococcus aureus*
